# Current State of Application of Machine Learning for Investigation of MgO-C Refractories: A Review

**DOI:** 10.3390/ma16237396

**Published:** 2023-11-28

**Authors:** Sebastian Sado, Ilona Jastrzębska, Wiesław Zelik, Jacek Szczerba

**Affiliations:** 1 Zaklady Magnezytowe “ROPCZYCE” S.A., Research and Development Centre of Ceramic Materials, ul. Przemysłowa 1, 39-100 Ropczyce, Poland; wieslaw.zelik@ropczyce.com.pl; 2 Faculty of Materials Science and Ceramics, AGH University of Kraków, al. A. Mickiewicza 30, 30-059 Kraków, Poland

**Keywords:** machine learning, MgO-C, refractory, steel, artificial neural networks, ANN

## Abstract

Nowadays, digitalization and automation in both industrial and research activities are driving forces of innovations. In recent years, machine learning (ML) techniques have been widely applied in these areas. A paramount direction in the application of ML models is the prediction of the material service time in heating devices. The results of ML algorithms are easy to interpret and can significantly shorten the time required for research and decision-making, substituting the trial-and-error approach and allowing for more sustainable processes. This work presents the state of the art in the application of machine learning for the investigation of MgO-C refractories, which are materials mainly consumed by the steel industry. Firstly, ML algorithms are presented, with an emphasis on the most commonly used ones in refractories engineering. Then, we reveal the application of ML in laboratory and industrial-scale investigations of MgO-C refractories. The first group reveals the implementation of ML techniques in the prediction of the most critical properties of MgO-C, including oxidation resistance, optimization of the C content, corrosion resistance, and thermomechanical properties. For the second group, ML was shown to be mostly utilized for the prediction of the service time of refractories. The work is summarized by indicating the opportunities and limitations of ML in the refractories engineering field. Above all, reliable models require an appropriate amount of high-quality data, which is the greatest current challenge and a call to the industry for data sharing, which will be reimbursed over the longer lifetimes of devices.

## 1. Introduction

Magnesia–carbon refractories (MgO-C) belong to the most significant type of refractories for steel and iron industry devices. They thermally protect basic oxygen furnaces (BOFs), steel ladles, and electric arc furnaces (EAFs), and they are used in the production of special products, like purging shapes and taphole sleeves [1]. The wear of MgO-C refractories is caused mainly by the attack of metallurgical slag, the oxidation of C by oxygen or other oxidizing compounds, and the interaction with CO/CO_2_, which occur at temperatures of 1600–1750 °C [1]. Also, the thermomechanical impact, associated with thermal shocks and the turbulent flow of hot metal, significantly influences the MgO-C refractory service time [1]. The typical service time of the MgO-C lining in BOFs varies from around 2000 up to 10,000 heats or more, depending on the maintenance conditions [2,3,4]. In steel ladles, the differentiation in the service time is substantial, as the ladle campaign might be finished after 123–183 heats [5], 70–85 heats [6], or even after only 8–20 heats [7]. The refractory lining service time in EAFs is also highly differentiated. The typical EAF lining service time is 500–1000 heats. But, even in one steel plant, it can vary from approximately 500 heats up to 1200 heats [8]. Refractory wear generates high maintenance costs. The high costs derive not only from the purchase and replacement of new refractory products but also from work stoppage and urgent repairs. The recent significant progress in R&D activities has influenced the extended service times of heating devices (e.g., the service times of refractories in steel ladles increased from 128 to 157 heats via the optimization of the lining materials and service conditions) [9]. Another example is increasing the VOD (vacuum oxygen decarburization) ladle service time from 8.5 heats (2017) to 20 heats (2021) via the addition of ZrSiO_4_, which enhanced the mechanical and thermomechanical properties of MgO-C bricks [7].

Although MgO-C refractories have been used since 1950 in steel and refining plants [10], their service times are still being extended owing to progressing research efforts. One of the main research directions in MgO-C improvement is the application of various metallic and non-metallic additions (e.g., Al, Mg, Si, SiC, Al-Mg, Fe) [11,12,13,14,15], as well as the development of new ones (e.g., c-ZrN nanopowder, Ti_3_AlC_2_, Ti_3_SiC_2_, Cr_3_C_2_C, spinel micro-powder, YAG nanopowder, and other oxide composites) [16,17,18,19,20,21,22,23,24,25]. Their addition to MgO-C improves decarburization resistance as well as hot properties, like hot strength and thermal shock resistance. The corrosion resistance of MgO-C has also been broadly investigated, with multiple techniques used, like the induction furnace test [26,27,28], finger test [29,30], sessile-drop technique [31,32,33], cup test [34,35,36,37], single hot thermocouple technique [38], and observations of in situ changes using a high-stage microscope [39]. Recently, new calcium–magnesium–aluminate raw materials have been developed, which promote the formation of a protective layer at the hot face of MgO-C bricks during operation [40,41,42]. With the increased demand for widely understood decarbonization and sustainable development, much effort is also being put into the recycling of MgO-C materials [43,44,45,46]. Ludwig et al. [43] obtained satisfactory results for 20% and even 30% additions of recycled MgO-C aggregate in a composition of new MgO-C brick. These areas have great potential for further improvements and research, as around 28 million tons of spent refractories are generated annually [45], while the total worldwide production of refractories is 35–40 million (70% for the steel industry).

However, the commonality in all these experimental studies is that both the experiment and result interpretations are always conducted in a traditional way, with a relatively low quantity of data taken for analysis. For MgO-C refractories, researchers are focused on very detailed investigations of the mechanisms responsible for the particular hot-temperature behavior of MgO-C bricks. Simultaneously, researchers have to face the high quantity of various data types [47]. For this reason, the refractory industry should take the opportunity of the available data and introduce techniques that allow for their better usage.

Recently, more companies have become interested in collecting data and finding relationships with refractories’ wear rates to optimize the process and make it more efficient as well as environmentally friendly. The implementation of Industry 4.0 [48] has created a new reality for many companies. This strategy has blurred the difference between the work of people and machines [48]. One of the objectives of Industry 4.0 is to achieve a higher level of digitalization and automation of and improvement in decision-making processes with automated data exchange [49]. An invaluable tool is machine learning (ML), the outstanding performance of which has so far been reported in numerous materials science studies [49]. ML algorithms refer to computational systems that can be trained to perform specific tasks, with no need to implement any explicit programming. Moreover, the quality of the algorithms’ performance improves with extended experience [49]. Interest in using ML techniques is constantly growing. The Web of Science database, when searched with the keyword “machine learning” 10 years ago (2013), showed 1908 papers, while, in 2022, 2021, and 2020, it showed 34,934, 30,053, and 22,335 papers, respectively. This 56% increase in the number of publications over the last 2 years and the 18-fold increase over the last 10 years permit the prediction of a forthcoming boom in ML utilization. Furthermore, data in the global datasphere are predicted to reach 175 zettabytes by 2025 (33 zettabytes in 2018) [50]. 

According to Pilania’s work [51], ML algorithms can be applied in various applications in materials science. One of its applications is the development of efficient and surrogate models which map and find relationships between a material’s composition, structure, morphology, and processing to select properties or performance criteria. Moreover, the author indicates numerous other fields of machine learning applications, like material characterization and design, designing of experiments, prioritizing of experiments, property prediction, and molecular and atomistic simulations [51].

Taking into account the relatively newly applied ML techniques in the refractories field and their vast innovation potential, this work aims to evaluate the most important published works on the application of various machine learning techniques in the investigation of MgO-C refractories. This review is divided into three main parts. Firstly, in Section 2 we present the most commonly applied ML algorithms and their utilization in different fields. Then, the current state of ML application in laboratory-scale examinations (Section 3) and in industrial-scale tests (Section 4) is revealed. The laboratory-scale works focus on the most critical properties of MgO-C refractories, including oxidation mechanisms, optimization of carbon content, corrosion resistance, and thermomechanical properties. The industrial-scale tests are aimed at the prediction of the service time of refractories in industrial heating devices. Finally, we summarize by indicating the benefits and limitations of ML utilization in research practice (Section 5). This work aims to be a reference for researchers who are searching for new capabilities and techniques to improve R&D activities in the technology of MgO-C refractories. 

## 2. Machine Learning Algorithms—An Overview 

**Figure 1:**
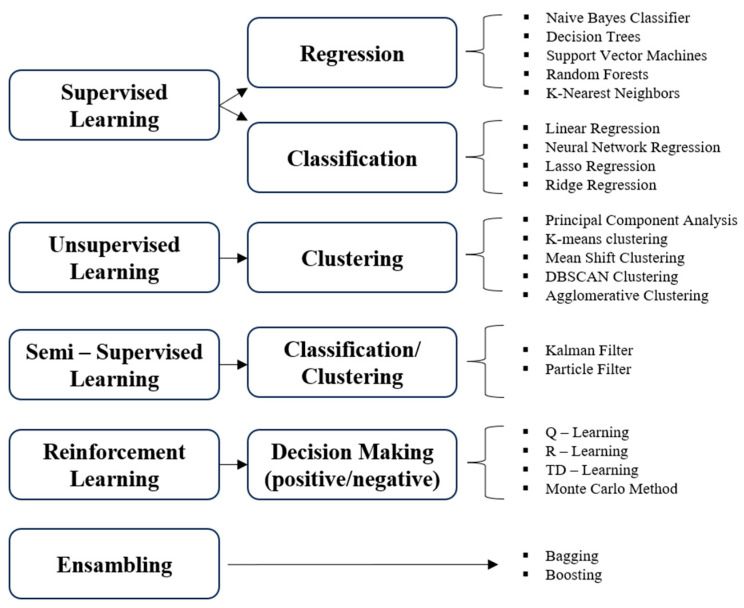
Overview of commonly used ML algorithms.

Machine learning is a subset of artificial intelligence. Algorithms are dedicated to building computational tools that make decisions without explicit coding. One of the main aims of the application of ML algorithms is taking the historical data and training the algorithms to further use these data in the prediction of specific features. The main advantage of ML algorithms is their powerful performance and speed of data processing compared to hand-coding. ML algorithms have proven their performance and utility in a variety of fields, such as speech recognition, text mining, medicine, data analysis, aeronautics, data analysis, stock market analysis, and many others [52,53]. This wide range of applications is possible due to a variety of existing algorithms which are presented in Figure 1 based on [51,52,53,54,55] (the graph does not exhaust all currently used algorithms). 

Sarker [52] has divided ML algorithms into four groups, including supervised learning (algorithms: classification and regression), unsupervised learning (clustering), semi-supervised learning (classification and clustering, based on labelled and unlabeled data) and reinforcement learning (positive and negative). 

Jain and Kumar [53] described three groups of ML algorithms, indicating specific ones in each of the groups. The first group is supervised learning with classification (algorithms: naïve Bayes, decision trees, support vector machines, random forest, K-nearest neighbors) and regression (linear regression, neural network regression, lasso regression, ridge regression). The second group is unsupervised learning (principal component analysis, K-means, mean shift clustering, DBSCAN clustering, agglomerative clustering). The third group is reinforcement learning (Q-learning, R- learning, TD-learning and the Monte Carlo method). 

According to Sarker [52], the algorithms with the highest popularity index worldwide are assigned to the group of reinforcement learning, but their popularity decreased in 2020. Pugliese et al. [54] showed that, in 2021, the popularity index of reinforced learning was still the highest, while supervised and unsupervised learning popularity indexes were on a similar level. As Pugliese et al. explains in [54], the popularity of reinforcement algorithms (algorithms based on interactions with the environment) reflects their use to solve real-world problems in a variety of fields, such as game theory, control theory, operation analysis, information theory, simulation-based optimization, manufacturing, supply chain logistics, swarm intelligence, aircraft control, robot motion control, laparoscopic surgery, traffic forecasting service, smart cities development, etc. [55]. 

## 3. Machine Learning in Investigation of MgO-C Materials

### 3.1. Application of ML in Laboratory-Scale Examinations

#### 3.1.1. Oxidation Mechanism of MgO-C Refractories

Oxidation of carbon in MgO-C refractories, especially below 1400 °C, is one of the main problems in the application of these materials [56]. The decarburized part of the refractory is loose and porous; thus, the slag and hot metal can easily penetrate the matrix. The mechanism of oxidation is widely examined using traditional techniques [57,58,59,60,61,62]. The decarburization resistance of MgO-C refractories is mostly affected by graphite content and the overall compactness of the brick.

Artificial neural networks (ANN), which represent one of the supervised ML techniques (belonging to Supervised Learning—Regression, Figure 1), were used by Nemati et al. [63] to predict the oxidation behavior of MgO-C materials. The authors tested several MgO-C materials with different carbon contents. ANN was used to predict the activation energy of oxidation, effective diffusion coefficient, and diffusion activation energy of oxidation. An input variable was the weight loss of MgO-C materials at different temperatures depending on graphite content from 4.5% to 17%. The model was developed using a standard feed-forward backpropagation network with one hidden layer. Oxidation of carbon in MgO-C refractories was found to be driven mainly by diffusion. The ANN model was also utilized to predict the effective diffusion coefficient at different temperatures. The obtained results were of good quality of fit, expressed by the determination coefficient R^2^ in the range 0.986–0.999. Finally, the three-layer back propagation ANN model was used to predict the oxidation kinetics of MgO-C specimens based on their weight loss at different temperatures. The authors developed reliable models with excellent fit between experimental and calculated data. The oxygen diffusion was reported as responsible for carbon oxidation in MgO-C refractories.

The oxygen diffusion mechanism in MgO-C composites was also investigated by A. Nemati et al. [64] with the use of the ANN approach. The authors used a standard feed-forward backpropagation network with one hidden layer. For training purposes, the Bayesian regularization algorithm was used (Levenberg–Marquardt modified backpropagation algorithm). Training was conducted with the use of different numbers of neurons in the hidden layer to find the optimal architecture. The external dataset from other authors’ experiments was utilized in this study. Similar to previous work [63], the input variables were the carbon content in the materials, oxidation temperature, and weight loss of the MgO-C specimens. It was assumed that three mechanisms control the oxidation rate: chemical adsorption, diffusion, and chemical reaction. Calculations were performed for a wide range of MgO-C materials, with graphite content varying from 5% to 30%. The authors developed models which enabled prediction of the effective diffusion coefficient for selected materials with the R^2^ coefficient in the range 0.986–0.999, depending on the carbon content and temperature of the test. If only one diffusion mechanism occurred, the low-temperature diffusion activation energy of oxidation was predicted to be in the range 21.2–35.0 kJ·mole^−1^, depending on the carbon content. The high-temperature diffusion activation energy was predicted to be in the range 42.1–109.6 kJ·mole^−1^ depending on the carbon content. If three diffusion mechanisms occurred, the low-temperature diffusion activation energy of oxidation was also predicted to be 16.5–25.7 kJ·mole^−1^ depending on the carbon content. The high-temperature diffusion activation energy was predicted to be 31.3–219.7 kJ·mole^−1^ depending on the carbon content. Authors also confirmed that predicted data are comparable with experimental data obtained by other authors in [59,60,65] The increased temperature resulted in an activation energy drop due to the increased oxygen diffusion rate. It was also confirmed that oxygen diffusion through the pores is the most significant factor controlling the oxidation intensity of MgO-C material. 

In described works [63,64], the authors conducted advanced calculations to predict the oxidation kinetic parameters of MgO-C samples depending on the carbon content and test temperatures. However, it is necessary to extend this research and find if it is possible to apply ML techniques for predicting the oxidation behavior of MgO-C bricks including other parameters like the compactness of the bricks, as far more factors affect the decarburization resistance of MgO-C bricks. Also, a greater number of samples should be used to obtain more reliable ML results. 

#### 3.1.2. Optimization of Carbon Content in MgO-C Refractories

Graphite is a main source of carbon in MgO-C refractories, which, due to its low thermal expansion coefficient and poor wettability by slag, provides high slag corrosion resistance and good thermal shock resistance, respectively. However, too much carbon in the MgO-C composition leads to heat loss in industrial devices during operation due to the increased conductivity of MgO-C materials [66]. Greater amounts of carbon in the MgO-C composition decrease the hot strength and oxidation resistance of MgO-C refractories [66]. Therefore, the optimization of graphite content in MgO-C bricks is crucial.

Mazloom et al. [67] used artificial neural networks to optimize graphite content in MgO-C refractories. The work aimed to find the optimal ratio of graphite to resin to provide the highest possible compressive strength and minimize the apparent porosity of the materials. Overall, 25 formulations of MgO-C refractories were selected, which varied in the amount of resin (1.0–3.0%) and graphite (7.5–17.5%). In total, 100 specimens were prepared (four specimens for each of 25 formulations) for experimental testing. According to the obtained results, it was found that replacing magnesia powder with graphite leads to a decrease in compressive strength of up to 10% of the graphite content. If the graphite content was 12.5%, the compressive strength increased, but further increasing the amount of graphite to 15% and 17% caused a decrease in compressive strength. The experimental results showed that an increase in the content of synthetic resin was always associated with an increase in cold compressive strength. The compactness of specimens with fixed resin content (determined by open porosity measurements) decreased with increased graphite content to 15% in MgO-C, while, above 15% graphite, only a slight increase in apparent porosity was observed. The larger amount of synthetic resin was considered to reinforce the effect of increased graphite content. For ML model development, backpropagation of training error and a three-layer network for training were used. Two variables were selected as input data, namely, resin and graphite content, while the output variables were ultimate compressive strength and apparent porosity of 100 specimens prepared experimentally. Approximately 250 cycles of training were conducted with the use of different numbers of neurons to find the best model. Applying ANN, it was reported that 13.5% graphite and 3.0% synthetic resin in formulation provide the highest ultimate compressive strength with the lowest apparent porosity. The model was validated experimentally on seven specimens, based on the ANN-proposed formulation. The ultimate cold compressive strength predicted by ANN was 365.16 MPa, while the experimental value was 376.47 MPa, which means a 1.3% error. ANN predicted an apparent porosity of 7.08% while the experimental was 7.11%, which gives a 0.35% error. The obtained results are shown in Table 1. As the authors stated, a reliable and accurate model is feasible to develop using ANN to predict the MgO-C material’s properties.

In [67], the authors determined the optimal amount of graphite and resin (13.5% graphite, 3% resin) to provide the demanded corrosion resistance with no loss in mechanical behavior. The authors show the prediction accuracy for selected specimens together with their open porosity and compressive strength. The accuracy of prediction is high (prediction error 1.30%), and the results of prediction seem to almost ideally fit the data. Therefore, it is good practice to describe the procedure applied to avoid overfitting (algorithmic learning by heart). Apparent porosity and the compressive strength of the samples were measured only on two specimens (out of each 25 formulations); thus, the results of measurements may not be reliable and need to be extended. Also, it would be recommended to investigate the influence of different raw material compositions together with different carbon and resin contents when modelling the basic properties of specimens. After all, the developed model is a useful tool to model and design MgO-C bricks with desirable properties.

#### 3.1.3. Corrosion Resistance of MgO-C Refractories

The corrosion resistance of refractory materials is widely studied due to its exceptional importance for the service life of heating devices. Corrosion of MgO-C refractories mostly limits the duration of a campaign of devices, which increases maintenance costs for the end users of refractories.

Optimization of MgO-C refractories’ composition for improved corrosion resistance was studied with unsupervised learning techniques using clustering algorithms [68]. A total of 20 different variants of MgO-C materials were prepared based on four different main raw materials. From each of the variants, eight industrially produced MgO-C bricks were selected for further examinations. Basic physicochemical properties (apparent porosity and bulk density, apparent porosity and bulk density after coking, decarburization resistance at 900 °C and 1100 °C, chemical composition, and graphite content) were experimentally measured. Principal component analysis (PCA) and the K-medoids algorithm were applied to develop a model which clusters the MgO-C materials into groups of comparable properties. PCA analysis showed that it is possible to use two variables, instead of eight, to characterize the prepared MgO-C materials. A new variable, PC1, was obtained, which explained approximately 81% of the variability in the dataset and referred to the basic properties of MgO-C materials. The second variable, PC2, explained about 12.3% of the data variability and referred to the values of pressure used for shaping the materials. In the K-medoids algorithm with PAM (partitioning around medoids), PC1 and PC2 were used as input variables. The algorithm was able to distinguish nine groups with materials of considerably comparable properties. It was assumed that materials assigned to the same clusters by the PAM algorithm have comparable corrosion resistance. Experimental tests of corrosion resistance were conducted with the use of an induction furnace to verify the obtained ML results. The algorithm indicated that a material consisting of fused magnesia of standard quality (shaped at 120 MPa) should perform similarly to a material consisting of 65% sintered and 12% fused magnesia of the highest quality (shaped at 180 MPa). Moreover, the algorithm suggested that materials composed of fused magnesia of standard quality and 27% sintered magnesia (shaped at 180 MPa) should perform similarly at high temperatures to test materials containing of 65% sintered magnesia and 12% of the highest-quality fused magnesia. With statistical tests (Wilcoxon test) applied to the measured wear rates after corrosion tests, it was confirmed that the described material variants were located in the same cluster indicated by the PAM algorithm and performed similarly after being exposed to slag attack at high temperature. Therefore, the algorithms properly indicated materials of comparable corrosion resistance. The conducted examinations, coupled with computer calculations, show the directions and possibilities to substitute fused raw materials with sintered ones with no loss of corrosion resistance. 

The described research [68] reveals an extremely important issue in terms of sustainable development, as the production of fused magnesia demands 15 times more energy than the production of sintered aggregates [69]. Even if the corrosion of MgO-C refractories is widely described in the literature [27,28,29,30,31,32,36,37,38,39,70,71,72], scarce information can be found regarding the comparison of high-temperature properties of MgO-C materials based on different magnesia raw materials. Using ML techniques, it was possible to group MgO-C compositions with different ratios of sintered to fused magnesia characterized by comparable corrosion resistance. The results were validated at a semi-industrial scale by conducting corrosion tests in an induction furnace at 1720 °C. This could contribute to extending the corrosion test to a wider temperature range of 1600–1750 °C, as impurities in magnesia raw materials affect MgO-C materials’ performance. Above all, it would be beneficial to test the designed materials in industrial conditions to assess the MgO-C materials’ real performance, e.g., in steel ladles which are typically characterized by the shortest service time of refractory lining.

The slag corrosion resistance of MgO-C refractories was also examined by Akkurt [73] with the use of artificial neural networks. The work aimed to predict the wear rate of MgO-C refractories for steel ladles based on the results of laboratory corrosion tests. The data were collected from a series of corrosion finger tests (without rotating the samples). The architecture of the designed ANN was as follows: three layers of the feed-forward type and six neurons in the input layer. The input variables were the percentage content of CO in the atmosphere, the time of brick exposure for slag attacks, the temperature of a test, and the CaO/SiO_2_ ratio of the slag. The measured surface of the lost area in the cross-section of tested specimens was taken as the output data. In the testing stage, the average testing error was reported at 14.2% with R^2^ = 0.92. A detailed comparison of predicted and experimental data is presented in Table 2. Surface plots showing the relationship between input variables and the percentage of lost area during corrosion were generated as a complementation of the results. It was shown that an increase in temperature lead to an increase in refractory wear. An insignificant interaction was observed between temperature and the time of lining exposure for corrosive factors. Some values of prediction error in Table 2 exceed 15%. This phenomenon is probably associated with the relatively low number of experimental measurements done by the authors; however, the results of ANN performance are consistent with the current state of knowledge concerning the MgO-C corrosion mechanism.

The conducted calculations are important to model process parameters in steel plants (e.g., % CO, temperature, heat time, slag basicity) and to provide appropriate corrosion resistance for refractories. It is worth noting that only seven observations (measured area loss) were used for model testing. Probably, the low number of measurements (both in the training procedure and testing) is the reason why the percent prediction error is high for some observations (23.4%, 34.3%). The presented research included only a few factors influencing the refractories’ corrosion resistance. If the model is allocated for direct use in steel plants, more factors should be included, e.g., number of heats/day, slag chemistry, tapping temperatures, secondary treatment temperatures, types of additives used for refining, etc. Moreover, a greater number of samples should be tested to provide higher accuracy of the model [74].

#### 3.1.4. Thermomechanical Properties of MgO-C Refractories

MgO-C refractories are exposed to extreme thermal, mechanical, and chemical stresses during operation in the steel plant. The highest thermal stresses occur during the preheating stage of the heating device and the tapping of the hot molten steel into the ladle, where the refractory lining suffers mostly from a high temperature gradient (from about 300 °C at the shell to 1600–1700 °C at the lining). For some applications, MgO-C materials have to withstand additional mechanical stresses, e.g., due to rocking of the BOF vessel during preheating [75,76].

An advanced investigation of the thermomechanical behavior of different lining concepts in steel ladles was conducted by Hou et al. [77]. Artificial neural networks were used, among others, to predict the thermal and thermomechanical responses of refractory lining during operation. Overall, 160 different configurations of lining were investigated. In this research, the finite element (FE) method was used to obtain the input data for the ANN architecture design. The FE calculation included preheating of the refractory bricks’ hot face in the ladle to 1200 °C for 20 h and direct exposure for tapping temperatures up to 1600 °C. A 95 min refining process was assumed. For the experiment and calculation, 10 different variables were used, assuming various steel shell lining thicknesses (insulation, permanent, and working linings), different thermal conductivities, and different Young’s modulus values for the bricks. Three-layer backpropagation ANN was used for prediction. Hyperbolic tangent sigmoid was selected as an activation function. Three tests were used to establish the optimal ANN architecture. At the first test, all 160 samples were selected for the training, where gradient descent with the adaptive learning rate backpropagation (GDX) algorithm was selected. In the second test, the data set was divided into three groups (96, 126, and 160 samples) to find the minimum sample size for the study. In the third test, eight different algorithms were used to find the most favorable one for the steel ladle. Model assessment was conducted with the use of various errors: RE_MAX (maximum relative error), MRE (mean relative error), RRMSE (relative root-mean-squared error), and coefficient of determination B. Out of eight algorithms, two were selected (CFG—conjugate gradient backpropagation with Fletcher–Reeves updating and BR—Bayesian regularization backpropagation) as the most suitable for calculations. The ANN was then built to compare the performance of the selected algorithms in the prediction of the end temperature (the temperature at the cold end of the steel shell), maximum tensile stress, and maximum compressive stress. The comparison results are shown in Table 3. Low values of RE_MAX and MRE and high values of B are desirable. For the maximum tensile strength and maximum compressive strength, the BR model performed more efficiently than the CFG model (for BR: higher values of the coefficient of determination B, lower values of MRE, and a lower value of RE_MAX for tensile strength). Based on the obtained results, the BP-ANN model with BR was utilized for final calculations.

The optimal ANN architecture was found for seven nodes in the hidden layer and Bayesian regularization with 160 samples for training. Two insulation lining concepts (linings 1 and 2 according to Table 4) were compared with the use of optimized ANN architecture. For this lining concept, predicted (ANN) and simulated (FE modelling) values of selected properties (steel shell temperature, maximum tensile stress, and maximum compressive stress) were shown in Table 5.

The results presented in Table 5 confirm that ANN performed outstandingly. The predicted values of selected properties (steel shell temperature, maximum tensile stress, maximum compressive stress) were close to the FE-simulated ones. The temperature difference between the predicted value and the value obtained through FE modelling for lining concept 1 was only 4 °C. Furthermore, for lining concept 2, the model predicted the same temperature of a steel shell as modelled through FE, 259 °C. The predicted maximum tensile stress for lining concept 1 was 1433 MPa, while it was 1495 MPa for FE modelling, which is a 4.1% error. For lining concept 2, the predicted maximum tensile stress was equal to 1576 MPa, while for the FE model it was 1539 MP, which is a 2.4% error. As for compressive stress for lining concepts 1 and 2, the differences between the predicted and FE-modelled maxima were 5 MPa and 2 MPa, respectively. The presented model was also reported as promising for material recipe improvements and steel production optimization.

The variation in the study [77] was analyzed in [78] to optimize the number of nodes used in the hidden layer of ANN. The Taguchi method was used to find the minimum numbers of input variables. Variation/response complexity was found to be crucial for establishing well-performed ANN architecture. The developed methodology and models were used to investigate higher numbers of lining concepts (192 linings) in the case of thermomechanical response in steel ladles [79].

The conducted calculations [77] have significant practical meaning in the case of assessing the thermomechanical behavior of ladle linings during operation. Based on 160 different lining concepts, two of them were selected, and their performance was adequately predicted using the ANN algorithm. The tapping temperature of molten steel was assumed to be 1600 °C, but it would be interesting for scientists and engineers to see the behavior of lining concepts up to 1700 °C. Even though the presented model has an indisputable influence on reducing the time, materials, and cost-consuming labor for trial on site, industrial verification should be done to investigate the designed lining performance under real conditions. Then, the models could be successfully used in industrial practice to design linings with appropriate thermal and thermomechanical properties. The presented work may not only affect the lining design but may also indicate directions for MgO-C materials or safety lining composition development. Furthermore, thermal and thermomechanical linings’ response may make it possible to avoid one of the most common failures of ladles associated with MgO-C thermal behavior (vertical cracks) [80].

## 4. Application of ML in Industrial-Scale Examinations

From the industrial point of view, the most important thing is to provide the longest possible service time for refractories in heating devices, which allows for the optimization of the cost-to-service time ratio. The service time of refractories is affected by several factors, including metallurgical conditions, refractory brick quality, the maintenance schedule of devices, etc. The service time is difficult to assess and predict. However, it seems to have become more feasible with the implementation of computational technologies in the development of refractories.

Borges et al. [81] applied self-organizing maps (SOM), which represent one of the unsupervised algorithms, to identify the main factor influencing the wear rate of MgO-C materials at the slag lines of the steel ladles. Around 6700 data points collected from the industrial database were analyzed. The authors compared the results of the traditional statistical approach with the SOM results. SOMs consisted of seven neurons vertically and six neurons horizontally for the selected properties. Approximately 23 metallurgical parameters were investigated. The SOM maps showed the relations between ladle service time and hot metal treatment with CaSi, Ar bubbling without CaSi, Ar bubbling with CaSi, steel permanence time, steel temperature after tapping, steel weight, and type of product (thick plates, hot strips, and boards for sale). At each step of the analysis, the results were verified with the use of typical regression and correlation analysis. Based on the SOM results, the authors indicated the numerous reasons responsible for the premature or intense wear of MgO-C materials in steel ladles, including the number of chemical additions (like nepheline and CaSi), the interaction between the desulphurization route and the intensity of ladle furnace use, and the extended contact time of the refractories with slag. The ML algorithm results were found to agree with traditional statistics calculations. The important fact in the described work [81] is that the authors verified the calculation results vs. traditional statistics and *post-mortem* results for selected MgO-C bricks. SOM maps are considered a useful tool, not only to indicate the parameters affecting ladle service time, but, thanks to the applied technique, direct recommendations may be allocated for steelmakers to improve the production process and extend the ladles’ performance. Considering industrial practice, it would be valuable to investigate not only the metallurgical factors but also the type of ladle maintenance (e.g., using gunning mixes or other protection techniques).

Another industrial study concerning ladle treatment was conducted by Jančar et al. [82]. The authors used selected metallurgical parameters as an input variable to build an artificial neural network for the prediction of ladle service time depending on input values. The output variable was the number of castings. For the calculations, data associated with the secondary treatment of a 230 t ladle in the steel plant Liberty Ostrava was used. 106 ladle campaigns were analyzed. At the first stage of analysis, a statistical evaluation of metallurgical parameters was performed. Parameters which insignificantly influenced data set variability were discarded from further analysis. For building the ANN, seven parameters were selected as input variables, namely, empty ladle time, full ladle period, tapping temperatures, steel temperature after tapping, electricity consumption, number of heats with vacuum treatment, and Ar consumption. A high-quality model was obtained during the network training, with a coefficient of determination R^2^ of 0.8927 for analyzing predicted vs. actual values of model output. During testing of the model, the coefficient of determination for actual and predicted ladle service times was R^2^ = 0.66. The developed model was used to simulate the ladle service time for a wide range of values of selected properties (time of full ladle, Ar consumption, tapping temperature, and electrical energy consumption). The authors showed that if the tapping temperature increases the ladle service time decreases (R^2^ = 0.9719), and, similarly, if electrical energy consumption increases during secondary treatment the service time of the ladle decreases (R^2^ = 0.9860). According to the presented studies, the authors show that, if the time of the full ladle (the overall time when metal is present in the ladle) increases, the ladle service time increases (R^2^ = 0.9249); similarly, if Ar consumption increases, the ladle service time increases (R^2^ = 0.9945). Furthermore, the authors extracted the selected variables’ importance and their influence on ladle service time using a developed criterion. Among the seven selected variables, the most negative impacts on ladle service time were from the time with an empty ladle (assumed importance coefficient of −21.08%), electrical energy consumption (−20.43%), and the number of heats with vacuum treatment (−15.71%). The most positive influences on ladle service time were associated with a higher amount of consumed Ar (+15.20%) and a longer time with the ladle filled with metal (+9.71%). Based on the obtained models [82], the authors expect to achieve increased ladle service times. Nevertheless, the authors will collect more data to expand their research.

The authors of [82] used a large industrial dataset collected from the steel production processes of 106 campaigns to develop a model predicting steel ladle service time. The authors were able to successfully indicate parameters which significantly influence ladle service time (tapping temperatures, energy consumption, time with a full ladle, Ar consumption, etc.). Taking into account overall ANN model performance, the tested model accuracy was not at a high level (R^2^ = 0.66). A probable reason for this is associated with the types of MgO-C materials installed in the ladle. As the authors indicated [82], 106 ladles were investigated, which were lined with different MgO-C grades. Information about the material’s quality should be used in further models as another input variable. Except for that fact, the presented work has significant practical meaning and the potential to be implemented in direct use at steel plants. The developed model could support steelmakers in the optimization of the process to provide safer work and higher performance for MgO-C refractories in steel ladles.

Yemelyanov et al. [83] proposed the use of artificial neural networks to diagnose lining conditions based on refractory lining thermograms. Work [83] showed detailed steps in conducting image recognition using an ANN. A method of preprocessing the thermal images was given by the authors to provide the best possible quality of data for input variables in the ANN. The input parameters were as follows: the mass centers of the thermograms, distance matrixes defining the borders of specific lining zones, and colors spotted on thermograms. The ANN was used in this work to classify the burnout zones of the lining. The training of the network was conducted in two steps. The first step was the typical training of the network with data sampling. In the second step, only experimental data were utilized for training. A total of 480 images of steel ladles and torpedo cars were applied for training. In the second stage, experimental thermograms (620 images) obtained from Alchevsk Iron and Steel Works were examined. The authors tested 22 neural networks to find the optimal architecture. The obtained results enabled them to implement specialized software in the steel plant that inspects the lining conditions.

It is worth emphasizing that the authors used unprecedented real thermograms of the lining collected at the steel plant. In total, 480 standard thermograms and 620 collected experimentally were used, which made it possible to obtain a reliable model with low values of classification error (0.258–0.443). The model performance was satisfactory and was the basis for developing specialist software for lining condition diagnosis. The major advantage of this work is that its results are positively implemented in industry. Moreover, it seems that the model is flexible and can be used successfully in various types of devices, e.g., steel ladles and torpedo ladles.

Zelik et al. [84] showed the application of artificial neural networks to predict the wear rate of MgO-C refractories in the slag spout zone of a basic oxygen furnace. One campaign of BOF was considered in the analysis. Overall, 17 variables, collected automatically at the steel plant, were assigned as input variables, including the chemical composition of hot metal, treatment temperature, the types of additive used in the process, and the type of maintenance operation (gunning and slagging). The residual thickness of the MgO-C bricks in the slag spout zone was taken as the output data. Measurement of the residual thickness of the bricks was conducted with the use of a laser scanner directly at the steel plant. The wear indexes were calculated based on 16 laser measurements of the lining. The values of residual thicknesses were divided into wear classes calculated according to Equation (1):

(1) $$up=t\cdot \frac{w}{10}$$

where *up* is the upper boundary of the wear class, *t* is the class number (1…10), and *w* is the maximum value of the wear index. ANN was used to predict the wear class depending on selected metallurgical parameters. The quality of training was 64.56% and 66.21% with testing performed using the R programming language. Table 6 presents the results of classification using the ANN model. Model performance was verified with the use of Orange 3.21 software. In this case, the classification accuracy reached 63.9%. Evaluation of the variables’ importance was done with the use of the Boosted Trees algorithm. The variables influencing the wear rate of MgO-C refractories most significantly were reported accordingly: the number of gunning operations was the most important, then the MgO content in the slag, the amount of lime added to the metal bath, and hot metal weight.

An extension of this work [84] was shown in [85]. The authors used industrial data on the metallurgical process in BOF to predict the wear rate of MgO-C refractories. A total of 13 variables were selected, including Si and C content in the hot metal, the temperature and weight of the hot metal, oxygen activity in the metal bath, the temperature at the end of the refining, the amount of oxygen used during the upper blow, the amount of calcium added to the metal bath, the amount of MgO-containing additive, and the chemical composition of the slag. Data were inspected and prepared in detail to provide the best possible quality. Exponential smoothing was implemented to remove noise from the data. Several ML models were tested to select the most accurate one for prediction, including multivariate adaptive regression splines (MARS), classification and regression trees (CART), boosted trees, and artificial neural networks (ANN, multilayer perceptron, MLP type). Boosted trees were reported to be the most effective in the prediction of the wear rate of MgO-C refractories. A comparison of the model performance was expressed with the use of different statistical measures: SSE (error sum of squares), MSE (mean squared error), RMSE (root-mean-square error), R^2^ (coefficient of determination), MAPE (mean absolute percentage error), and MAE (mean absolute error), as shown in Table 7. This extended analysis made it possible to indicate parameters that significantly influence the service time of BOF. The most important factors were found to be hot metal weight, then the Si concentration in the hot metal, scrap mass, and the oxygen activity in the hot metal.

Two works [84,85] describe the application of different ML techniques for prediction of the wear rates of MgO-C materials in basic oxygen converters based on metallurgical parameters collected during hot metal treatment. Authors obtained models of different qualities. Among the used techniques, ANN and the boosted trees algorithm were reported as producing the most accurate results. Even though the works carry practical meaning, the model performance needs to be improved. The directions of model performance improvement are associated with the quality of the data. First of all, in both works [84,85], the residual thickness of MgO-C materials in the slag spout zone was used as an output variable. Unfortunately, due to the specific work of the steel plant, only about 20 laser-scanned results for lining thickness were obtained during the campaign. Such a low amount of data in the campaign, which lasts more than 2000 heats, affects the ML model’s quality. Industrial data are often not prepared well enough and contain missing or invalid values (e.g., the amount of hot metal exceeding the device’s capacity). Collecting quantitative data on the gunning mixes used for sidewall protection would also improve the quality of the models.

## 5. Benefits and Limitations of the Application of ML Techniques for the Investigation of MgO-C Refractories

Although the number of publications on the application of machine learning is rapidly growing, with a 56% increase over the last 2 years, it is still very low when it comes to ML application in the refractory industry. Based on reviewed works [63,64,67,68,73,77,78,79,81,82,83,84,85], the most commonly used ML algorithm, and simultaneously the one giving the most accurate predictions, is an artificial neural network. One exception is given in [85], which shows boosted trees are the best-fitting algorithm. The presented articles prove that ML algorithms are highly useful in examinations and in industrial applications of MgO-C materials. In the research process, the most significant advantage of applying ML algorithms is the reduction of time-consuming and expensive experimental investigations in the corrosion testing of MgO-C refractories [63,64].

ML techniques currently have obvious limitations, as the quality of data collected in the industry is still not satisfactory. Thus, it is necessary and highly recommended to improve the process of data registration, especially data involving steel production processes, to avoid missing data, unreal values, or mistake-generative hand typing. Using data of unsatisfactory quality may lead to inaccurate conclusions.

Another important limitation is related to laboratory experiments and the fact that ML algorithms are trained on data collected from specific, highly advanced examinations. It might be difficult to apply external data to such models and obtain reliable results, especially if one—allegedly insignificant—factor is changed.

Nevertheless, interest in using ML techniques in the refractory industry is growing, as it seems that high digitalization in this area is unavoidable. The possibility of predicting the wear rate of refractories depending on their metallurgical data will be especially encouraging for refractory end-users. They should be conscious of the need to improve data collection in order to develop highly predictive models that will serve in industrial practice and help to make the steel process more sustainable.

## 6. Conclusions

The current state of knowledge on ML techniques—relatively newly applied in refractories investigation—was reviewed in this work for MgO-C materials, which constitute over 70% of total refractories production. The most commonly used ML algorithm type is currently artificial neural networks. The clustering algorithm is also effectively applied in the optimization of MgO-C materials and the identification of factors influencing vessels’ service time in steel production.

Nevertheless, the number of papers on the application of ML techniques is still insufficient considering the rapidly growing interest in and high potential of ML techniques. The limited accessibility of reliable data is one of the reasons, which results from the disclosure politics of steel plants. The end users of MgO-C refractories will be conscious of the benefits gained from building high-quality ML models, which can influence the extension of the service time of refractories, thus making the steel production process more efficient and sustainable.

Concerning experimental research activities on MgO-C refractories, it is always cost-intensive to prepare and analyze the great number of samples demanded for ML implementation. The experimental approach has been changing, and wide implementation of ML in the refractory industry is unavoidable to speed up innovation in the industry in the near future, which stands at the front of a fast-changing and challenging environment.

## Figures and Tables

**Table 1:** Experimental results for the compressive strength and apparent porosity of samples based on the optimum formulations from the ANN model [67].

Sample	Compressive Strength [MPa]	Apparent Porosity [%]
F1	381.20	-
F2	375.91	-
F3	371.25	-
F4	377.54	-
F5	-	7.05
F6	-	7.18
F7	-	7.09
Average experimental value	376.47	7.11
Predicted value (ANN)	365.16	7.08
Error, % *	1.30	0.35

* difference between experimental and predicted values.

**Table 2:** Results of model testing, based on [73].

% Area Loss— Measured	% Area Loss— Predicted	Difference	% Error (Absolute)
10.57	12.43	−1.86	17.6
10.85	14.57	−3.72	34.3
14.65	14.85	−0.20	1.4
18.99	18.94	0.05	0.3
19.20	18.05	1.15	6.0
32.34	24.80	7.54	23.3
15.67	18.27	−2.60	16.6
Average	-	-	14.2

**Table 3:** Thermomechanical response prediction with the use of ANN based on CGF and BR, based on [77].

	End Temperature [°C]		Maximum Tensile Stress [MPa]		Maximum Compressive Stress [MPa]	
Used algorithm	CFG	BR	CFG	BR	CFG	BR
RE_MAX [%]	7.15	7.15	16.62	12.43	3.12	4.09
MRE [%]	1.02	1.76	2.43	2.37	0.93	0.78
B	0.9967	0.9908	0.9279	0.9348	0.9963	0.9966

RE_MAX, MRE, and B—coefficients evaluating the error between the results of the two used algorithms, CFG and BR.

**Table 4:** Refractory lining concepts selected for prediction, based on [77].

	Thickness [mm]	Thermal Conductivity [W·m^−1^K^−1^]	Young’s Modulus [GPa]	Thermal Expansion Coefficient [10^−6^K^−1^]
Working lining	155.0	9	40	12
Permanent lining	52.5	2.2	45	5
Insulation (lining concept 1)	37.5	0.5	3	6
Insulation (lining concept 2)	37.5	0.38	4	5.6
Steel shell	30	50	210	12.0

**Table 5:** Comparison of simulated and predicted values of two proposed optimal lining concepts from FE modelling and as predicted by BP-ANN, based on [77].

	Steel Shell Temperature [°C]		Maximum Tensile Stress [MPa]		Maximum Compressive Stress [MPa]	
	Modelling (FE)	Predicted (BP-ANN)	Modelling (FE)	Predicted (BP-ANN)	Modelling (FE)	Predicted (BP-ANN)
Lining concept 1	280	276	1495	1433	512	517
Lining concept 2	259	259	1539	1576	517	515

**Table 6:** Classification of wear rate class conducted with the use of ANN model, based on [84].

**Predicted Wear Class**	**Real Wear Class**											
		**0**	**1**	**2**	**3**	**4**	**5**	**6**	**7**	**8**	**9**	**∑**
	**0**	226	60	19	0	4	0	1	0	0	0	310
	**1**	63	128	2	0	4	0	12	0	0	0	209
	**2**	9	1	6	0	0	0	0	0	0	0	16
	**3**	0	0	0	0	0	0	0	0	0	0	0
	**4**	12	12	10	0	0	0	7	0	0	8	49
	**5**	0	0	0	0	0	0	0	0	0	0	0
	**6**	0	6	0	0	5	0	5	0	0	0	16
	**7**	0	0	0	0	0	0	0	0	0	0	0
	**8**	0	0	0	0	0	0	0	0	0	0	0
	**9**	0	10	0	0	6	0	2	0	0	7	25
	**∑**	310	217	37	0	19	0	27	0	0	15	625

**Table 7:** Comparison of different measures of fit for model performance, based on [85].

**Training Data Set**							
Algorithm	SSE	MSE	RMSE	R^2^	R	MAPE	MAE
CART	6.811	0.004	0.065	0.559	0.747	24.673%	0.057
MARS	4.195	0.002	0.051	0.716	0.846	17.987%	0.047
Boosted Trees	1.590	0.001	0.031	0.899	0.948	11.086%	0.029
ANN	3.521	0.002	0.047	0.789	0.886	16.012%	0.041
**Testing Data Set**							
CART	5.445	0.008	0.091	0.429	0.655	27.598%	0.066
MARS	3.329	0.005	0.071	0.649	0.805	21.316%	0.054
Boosted Trees	1.458	0.002	0.047	0.849	0.921	13.439%	0.035
ANN	2.932	0.004	0.066	0.687	0.829	20.233%	0.049

## Data Availability

Data are contained within the article.
